# Association Between State Opioid Prescribing Limits and Duration of Opioid Prescriptions From Dentists

**DOI:** 10.1001/jamanetworkopen.2022.50409

**Published:** 2023-01-11

**Authors:** Kao-Ping Chua, Thuy D. Nguyen, Jennifer F. Waljee, Romesh P. Nalliah, Chad M. Brummett

**Affiliations:** 1 Susan B. Meister Child Health Evaluation and Research Center, Department of Pediatrics, University of Michigan Medical School, Ann Arbor; 2 Department of Health Management and Policy, University of Michigan School of Public Health, Ann Arbor; 3 Section of Plastic Surgery, Department of Surgery, University of Michigan Medical School, Ann Arbor; 4 Michigan Opioid Prescribing Engagement Network, Institute for Healthcare Policy and Innovation, University of Michigan Medical School, Ann Arbor; 5 University of Michigan School of Dentistry, Ann Arbor; 6 Division of Pain Medicine, Department of Anesthesiology, University of Michigan Medical School, Ann Arbor

## Abstract

**Importance:**

In part to prevent the harms associated with dental opioid prescriptions, most states have enacted policies limiting the duration of opioid prescriptions for acute pain. Whether these limits are associated with changes in the duration of opioid prescriptions written by dentists is unclear.

**Objective:**

To evaluate the association between state opioid prescribing limits and the duration of opioid prescriptions from dentists.

**Design, Setting, and Participants:**

This difference-in-differences cross-sectional study used data from the IQVIA Longitudinal Prescription Database, an all-payer database reporting prescription dispensing from 92% of retail pharmacies in the US. The sample included opioid prescriptions from dentists dispensed to children aged 0 to 17 years and adults 18 years or older from January 2014 through February 2020. Treatment states were those that implemented limits between January 2016 and December 2018. Control states were those that did not implement limits during the study period. Data on opioid prescribing limits were derived from the Prescription Drug Abuse Policy System. Data were analyzed from January 1 to September 30, 2022.

**Exposures:**

State opioid prescribing limits.

**Main Outcomes and Measures:**

The outcome was opioid prescription duration, as measured by days’ supply. The association between limits and duration was evaluated using a linear model with a 2-way fixed-effects specification. Covariates included patient characteristics, prescription characteristics, and indicators of implementation of prescription drug monitoring program use mandates. Separate analyses of data from adults and children were conducted owing to differences in the number of treatment states and restrictiveness of limits by age.

**Results:**

The adult analysis included 56 607 314 opioid prescriptions for 34 364 775 patients (18 448 788 females [53.7%]; mean [SD] age at the earliest fill, 44.0 [17.4] years) in 22 treatment states and 12 control states. The child analysis included 3 720 837 opioid prescriptions for 3 165 880 patients (1 740 449 females [55.0%]; mean [SD] age at the earliest fill, 14.4 [3.5] years) in 23 treatment states and 12 control states. In both analyses, the median (25th-75th percentile) duration of opioid prescriptions was 3.0 (2-5) days. Implementation of limits, most of which allowed up to a 7-day supply of opioids, was not associated with changes in the duration of opioid prescriptions for adults (mean days’ supply: −0.06 days; 95% CI, −0.11 to <0.001 days) or children (mean days’ supply: −0.07 days; 95% CI, −0.15 to 0.02 days).

**Conclusions and Relevance:**

In this study of national pharmacy dispensing data, opioid prescribing limits were not associated with changes in the duration of opioid prescriptions from dentists. Future research should investigate the potential role of alternative interventions in reducing opioid prescribing by dentists.

## Key Points

**Question:**

Are state opioid prescribing limits associated with reductions in the duration of opioid prescriptions from dentists?

**Findings:**

In this cross-sectional study of nearly 90 million dental opioid prescriptions dispensed to adults and children in the US from January 2014 through February 2020, the median duration of these prescriptions was 3.0 days. Implementation of state opioid prescribing limits, most of which allowed up to a 7-day supply of opioids, was not associated with changes in the duration of these prescriptions.

**Meaning:**

The findings of this study suggest the need for future research evaluating the potential role of alternative interventions in reducing dental opioid prescribing.

## Teaser

This cross-sectional study assesses whether state-specific implementation of opioid prescribing is associated with a shorter duration of opioid prescriptions from dentists for children and adult dental patients.

## Introduction

In 2019, US dentists provided 10.9 million opioid prescriptions, representing 7.6% of the opioid prescriptions dispensed that year.^1^ Exposure to dental opioid prescriptions is associated with an increased risk of persistent opioid use, addiction, and overdose.^2,3,4,5,6,7^ Moreover, the risk of opioid-related adverse events, such as persistent opioid use, increases as the duration of these prescriptions gets longer.^6^ Consequently, decreasing the duration of dental opioid prescriptions could play a role in preventing patient harm while reducing the number of leftover opioid pills available for misuse.

In part to achieve this goal, at least 39 states have enacted restrictions on the duration of opioid prescriptions for patients with acute pain, patients who are new to opioids, or both.^8,9^ Implementation of these limits could be associated with reduced duration of dental opioid prescriptions that otherwise would have exceeded the allowed duration. On the other hand, these limits may be associated with minimal changes if the duration was already below the maximum level allowed.^8,10^ To date, few national studies have evaluated these possibilities. In an analysis of Medicare claims, implementation of state opioid prescribing limits was associated with a 0.9-day decrease in the duration of opioid prescriptions from dentists and surgeons.^11^ However, that study did not separately analyze opioid prescriptions from dentists and did not include patients without Medicare coverage, who received 85% of opioid prescriptions from US dentists in 2019.^1^

The objective of this cross-sectional study was to evaluate the association between state opioid prescribing limits and the duration of opioid prescriptions from dentists. We hypothesized that limits would not be associated with reduced duration, as most limits allow for a 7-day supply of opioids and most dental opioid prescriptions are written for a 3-day supply.^12^

## Methods

### Data Sources

We analyzed data (2014 to 2020) from the IQVIA Longitudinal Prescription Database. This database captures 92% of prescriptions dispensed in US retail pharmacies (including chain and independent) and the majority of prescriptions dispensed in mail-order and long-term care pharmacies. Because the data used were deidentified, the University of Michigan Medical School Institutional Review Board deemed this difference-in-differences cross-sectional study exempt from review and waived the informed consent requirement. We followed the Strengthening the Reporting of Observational Studies in Epidemiology (STROBE) reporting guideline.^13^

During the study period, the degree of pharmacy coverage in the database remained stable, according to Allen Campbell, BS, at IQVIA (written/oral/email communication, November 2022). Data elements included patient demographic characteristics (eg, age, sex, and state of residence), prescription method of payment, prescriber specialty, and days’ supply. We obtained details on state limits from the Prescription Drug Abuse Policy System (PDAPS), an organization that conducts professional legal epidemiological research on substance use policies.^14^ We also used the PDAPS to obtain details on state policies mandating clinicians to review a prescription drug monitoring program (PDMP) database before prescribing controlled substances (ie, PDMP use mandate), a potential confounding policy.

### Study Sample

We initially included all opioid analgesic prescriptions from general dentists, dental subspecialists, and oral and maxillofacial surgeons that were dispensed from January 2014 through February 2020 to children aged 0 to 17 years and adults 18 years or older residing in 1 of the 50 US states or the District of Columbia. Prescriptions that were dispensed after February 2020 were not included to avoid potential confounding related to the COVID-19 pandemic. Among prescriptions meeting inclusion criteria, we excluded those with missing data for covariates or potentially invalid dosing information (<0 quantity, <0 days’ supply, >90 days’ supply).

We identified opioid analgesics using IQVIA’s market definition. This definition included liquid and transdermal opioid products but did not include opioid cough-and-cold medications or buprenorphine formulations used for opioid use disorder. The eAppendix 1 in Supplement 1 lists the opioid analgesics we analyzed.

### Study Design

We used a difference-in-differences approach. In this approach, the difference in mean outcomes between the preintervention and postintervention periods in the control group is subtracted from the corresponding difference in the treatment group. The resulting difference-in-differences estimate is only unbiased if the outcome in the treatment group would have trended in the same direction as the control group in the absence of the intervention. Confidence in this assumption is increased if the preintervention trends among the treatment and control groups are parallel.^15^

We conducted separate analyses for adults and children, as some states imposed opioid prescribing limits for only 1 age group and other states imposed limits that were more stringent for children than for adults. In both the adult and child analyses, the control states included 11 states (Alabama, California, Georgia, Idaho, Iowa, Kansas, New Mexico, North Dakota, Oregon, South Dakota, and Wisconsin) that did not enact an opioid prescribing limit from January 2014 through February 2020, as well as the District of Columbia, which also did not enact a limit during this period. To mitigate bias from staggered treatment timing and to capture sufficient preintervention and postintervention data, we allowed only those states that enacted limits between January 2016 and December 2018 to be the treatment states.

We excluded states that enacted limits during this period in the same month that they implemented or amended a PDMP use mandate for dentists because disentangling the implications of the limits from these other policy changes would be challenging. We also excluded states that only enacted limits for opioid prescriptions written under certain circumstances (eg, emergency department care). After these exclusions, there were 22 treatment states in the adult analysis and 23 treatment states in the child analysis; the notes for Table 1 and Table 2 provide further details.

**Table 1.** Opioid Prescribing Limits for Dentists in 22 Treatment States: Adult Analysis^a^

State	Limit implementation date	Maximum duration allowed, d	Type of opioid prescription	Definition of initial prescription	Professional judgment exception
Arizona	April 26, 2018	5	Initial prescription, Schedule II opioids	No opioid prescription within 60 d	No
Arkansas	June 28, 2018	7	Any prescription, Schedule II-III opioids	NA	No
Colorado	May 21, 2018	7	Initial prescription, all Schedule opioids	First prescription from prescriber to patient in past y	Yes
Connecticut	July 1, 2016	7	Initial prescription for outpatient use, all Schedule opioids	Not defined	Yes
Indiana	July 1, 2017	7	Initial prescription for outpatient use and acute pain, all Schedule opioids	First opioid prescription from prescriber to patient	Yes
Kentucky	June 29, 2017	3	Prescription for acute pain, Schedule II opioids	NA	Yes
Louisiana	August 1, 2017	7	Initial prescription for outpatient use for acute pain, all Schedule opioids	Not defined	Yes
Massachusetts	March 14, 2016	7	Initial prescription for outpatient use, all Schedule opioids	First opioid prescription from prescriber to patient	Yes
Michigan	July 1, 2018	7	Prescription for acute pain, all Schedule opioids	NA	No
Minnesota	July 1, 2017	4	Prescription for acute dental pain, Schedule II-IV opioids	NA	Yes
Mississippi	October 28, 2018	10	Prescription for acute pain, all Schedule opioids	NA	No
Missouri	August 28, 2018	7	Initial prescription for acute pain, all Schedule opioids	NA	Yes
New Jersey	March 1, 2017	5	Initial prescription for acute pain, all Schedule opioids^b^	Not defined	No
New York	July 22, 2016	7	Initial prescription for acute pain, Schedule II-IV opioids	No opioid prescription in past y	No
North Carolina	January 1, 2018	5	Prescription for acute pain, Schedule II-III opioids	Not defined	No
Ohio	August 31, 2017	7	Prescription for acute pain, all Schedule opioids	NA	Yes
Oklahoma	November 1, 2018	7	Initial prescription for acute pain	No opioid prescription in past y	Yes
Rhode Island	March 22, 2017	20 Doses	Initial prescription for acute pain	No opioid prescription in past y	No
South Carolina	May 15, 2018	7	Initial prescription for acute pain, all Schedule opioids	NA	No
Vermont	July 1, 2017	7^c^	Initial prescription, all Schedule opioids	≤7 Consecutive d of opioid therapy in previous 30 d	Yes
Virginia	March 24, 2017	7	Initial prescription for acute pain, Schedule II-IV opioids	First opioid prescription from prescriber to patient	No
West Virginia	June 27, 2018	3	All prescriptions for Schedule II opioids^d^	NA	No

Abbreviation: NA, not applicable.

^a^ The 12 control states were Alabama, California, Georgia, Idaho, Iowa, Kansas, New Mexico, North Dakota, Oregon, South Dakota, and Wisconsin plus the District of Columbia. A total of 4 states were excluded because limits were implemented in 2019 or afterward (Montana, Texas, Washington, and Wyoming). One state was excluded because its limit was implemented in 2012 (Illinois). Another 7 states were excluded because they implemented prescription drug monitoring program use mandates affecting dentists or amended an existing mandate in the same month as a limit (Alaska, Delaware, Florida, Maine, Nevada, Tennessee, and Utah). The following 3 states were not included as either a treatment or control state: Hawaii (implemented 7-day limit only for opioids overlapping with benzodiazepine prescriptions), Maryland (implemented a limit requiring the lowest effective dosage but no hard limit), and New Hampshire (implemented a law that affected opioid prescribing in only emergency departments, retail clinics, and urgent care centers). Pennsylvania was excluded because it implemented a limit for adults only in the emergency department, and Nebraska was excluded because its limit affected only children. After exclusion of these 17 states, 22 treatment states remained.

^b^ New Jersey amended the law on June 5, 2017, so that it only affected Schedule II opioids.

^c^ For Vermont, the limit varies based on severity of pain. The table lists the limit for the highest level of pain specified for adults (extreme).

^d^ West Virginia amended the law on June 7, 2018, so that it only affected Schedule II opioids.

**Table 2.** Opioid Prescribing Limits for Dentists in 23 Treatment States: Child Analysis^a^

State	Limit implementation date	Maximum duration allowed, d	Type of opioid prescription	Definition of initial prescription	Professional judgment exception
Arizona	April 26, 2018	5	Initial prescription, Schedule II opioids	No opioid prescription within 60 d	No
Arkansas	June 28, 2018	7	Any prescription, Schedule II-III opioids	NA	No
Colorado	May 21, 2018	7	Initial prescription, all Schedule opioids	First prescription from prescriber to patient in past y	Yes
Connecticut	July 1, 2016	7^b^	Any prescription, all Schedule opioids	Not defined	Yes
Indiana^c^	July 1, 2017	7	Any prescription, all Schedule opioids	First opioid prescription from prescriber to patient	Yes
Kentucky	June 29, 2017	3	Prescription for acute pain, Schedule II opioids	NA	Yes
Louisiana^c^	August 1, 2017	7	Any prescription, all Schedule opioids	Not defined	Yes
Massachusetts^c^	March 14, 2016	7	Any prescription, all Schedule opioids	First opioid prescription from prescriber to patient	Yes
Michigan	July 1, 2018	7	Prescription for acute pain, all Schedule opioids	NA	No
Minnesota	July 1, 2017	4	Prescription for acute dental pain, Schedule II-IV opioids	NA	Yes
Mississippi	October 28, 2018	10	Prescription for acute pain, all Schedule opioids	NA	No
Missouri	August 28, 2018	7	Initial prescription for acute pain, all Schedule opioids	NA	Yes
New Jersey	March 1, 2017	5	Initial prescription for acute pain, all Schedule opioids^d^	Not defined	No
Nebraska	July 19, 2018	7	Prescription for outpatient use for acute condition, all Schedule opioids	NA	Yes
New York	July 22, 2016	7	Initial prescription for acute pain, Schedule II-IV opioids	No opioid prescription in past y	No
North Carolina	January 1, 2018	5	Prescription for acute pain, Schedule II-III opioids	Not defined	No
Oklahoma	October 28, 2018	7	Initial prescription for acute pain	No opioid prescription in past y	Yes
Ohio^c^	August 31, 2017	5	Initial prescription for acute pain, all Schedule opioids	NA	Yes
Pennsylvania	February 4, 2017	7	Initial opioid prescription, all Schedule opioids	First prescription in a single course of treatment	Yes
South Carolina	May 15, 2018	7	Initial prescription for acute pain, all Schedule opioids	NA	No
Virginia	March 24, 2017	7	Initial prescription for acute pain, Schedules II-IV	First opioid prescription from prescriber to patient	No
Vermont^c^	July 1, 2017	3^e^	Initial prescription, all Schedule opioids	≤7 Consecutive days of opioid therapy in previous 30 d	Yes
West Virginia	June 27, 2018	3	All prescriptions for Schedule II opioids^f^	NA	No

Abbreviation: NA, not applicable.

^a^ The 12 control states (including the District of Columbia) in the child analysis were the same as in the adult analysis. Four states were excluded because limits were implemented in 2019 or afterwards (Montana, Texas, Washington, and Wyoming). One state was excluded because its limit was implemented in 2012 (Illinois). Seven states were excluded because they implemented prescription drug monitoring program use mandates affecting dentists or amended an existing mandate in the same month as a limit (Alaska, Delaware, Florida, Maine, Nevada, Tennessee, and Utah). Hawaii was excluded because it implemented a 7-day limit only for opioids overlapping with benzodiazepine prescriptions. Maryland was excluded because its limit required the lowest effective dosage, but it was not a hard limit. New Hampshire was excluded because it implemented a law that affected opioid prescribing to adults in only emergency departments, retail clinics, and urgent care centers. Rhode Island was excluded because its limit for children was implemented in July 2019, meaning it would not have sufficient postintervention data if included as a treatment state (the Rhode Island limit for adults was implemented in March 2017). After exclusion of these 16 states, 23 treatment states remained.

^b^ On July 1, 2017, Connecticut amended the limit so that all opioid prescriptions for minors were restricted to a 5-day supply rather than a 7-day supply.

^c^ State was included in the adult analysis but imposed more restrictive limits for children.

^d^ New Jersey amended the law on June 5, 2017, so that it only affected Schedule II opioids.

^e^ For Vermont, the limit for children varies based on severity of pain. The table lists the limit for the highest level of pain specified for children (moderate to severe).

^f^ West Virginia amended the law on June 7, 2018, so that it only affected Schedule II opioids.

### Outcome and Covariates

The outcome was the duration of opioid prescriptions, as measured by days’ supply. This outcome is directly reported in the IQVIA database and represents the number of days of therapy that is fully covered by a prescription if the maximum daily number of doses allowed is taken. For example, 24 opioid pills correspond to a 4-day supply if a prescription is written for 1 pill every 4 hours as needed for pain (ie, 6 doses per day).

Covariates included an indicator that equaled 1 if prescriptions were dispensed on or after the enactment date of a PDMP use mandate for dentists or 0 if otherwise (eAppendix 2 in Supplement 1). Covariates also included patient and prescription characteristics, including patient age and sex, prescription method of payment, prescriber specialty (oral and maxillofacial surgeon vs general or subspecialty dentist), US Drug Enforcement Administration (DEA) Schedule, and prior opioid use. Following a National Quality Forum–endorsed quality measure, prior opioid use was defined as any opioid dispensing in the past 90 days.^16^

### Statistical Analysis

To evaluate the association between opioid prescribing limits and the duration of opioid prescriptions from dentists, we fitted linear regression models with a 2-way fixed-effects specification. The model included fixed effects for state, fixed effects for year-month, variables for each covariate, and an indicator that equaled 1 if the prescription was dispensed on or after the limit implementation date minus 6 months or 0 if otherwise (primary variable of interest). We began the postintervention period 6 months before the limit implementation because the event study analysis suggested differential declines in days’ supply in the treatment states 5 months prior to the limit implementation in the adult analysis and 6 months prior to the limit implementation in the child analysis. These patterns suggested that dentists may have changed their prescribing behavior in anticipation of the limit enactment.

To assess whether the association between limits and duration varied by limit restrictiveness, we conducted 2 difference-in-differences analyses. First, to compare the association among states that restricted dental opioid prescriptions to a 5-day supply or less vs greater than a 5-day supply, we fitted the same model used in the main analysis but included an indicator that equaled 1 if the prescription was dispensed on or after the limit implementation date, minus 6 months, in a state that restricted prescriptions to a 5-day supply or less. For all other prescriptions, the indicator equaled 0. Second, we used a similar approach to assess whether the association varied among states that restricted opioid prescriptions to a 3-day supply vs greater than a 3-day supply.

To assess for variation in association by dental specialty, we fitted the same model used in the main analysis. We included in the model an interaction between the primary variable of interest and an indicator for whether the prescription was written by an oral and maxillofacial surgeon or by another type of dentist.

To assess the parallel trends assumption, we conducted an event study analysis. We fitted linear regression models with fixed effects for state, fixed effects for year-month, variables for each covariate, and dummy variables for the number of months to the intervention in treatment states. The reference period was the month before limit implementation minus 6 months. The number of leads and lags was capped to ensure that all treatment states contributed equivalent amounts of data, thus avoiding bias from compositional changes in the states contributing to each data point. The time caps were −16 months for leads and +16 months for lags, as 1 treatment state (Oklahoma) implemented a limit in November 2018, 16 months before the end of the study period in February 2020.

All models used robust SEs clustered at the state level. This approach accounted for the association between the limits and the duration of dental opioid prescriptions among patients in the same state. Analyses were performed with R, version 4.3.1 (R Foundation for Statistical Computing), and 2-sided hypothesis tests with α = .05 were used to determine statistical significance. Data were analyzed from January 1 to September 30, 2022.

In sensitivity analyses, we repeated the main analyses when assuming a 3-month anticipation period and no anticipation period. Additionally, we repeated the analyses when using doubly robust difference-in-differences estimation, which combines outcome regression adjustment with inverse probability weighting to reduce the bias that occurs in 2-way fixed-effects estimates when the magnitude of the association between the exposure and outcome changes over time.^17,18^ Finally, to explore whether the conclusions would change if alternative measures of dosing were used, we repeated the analyses when modeling total morphine milligram equivalents (MMEs), a standardized measure of opioid prescription size,^19^ and when modeling the proportion of opioid prescriptions exceeding a 5-day or 7-day supply.

## Results

### Adult Analysis

The IQVIA database included 85 632 541 dental opioid prescriptions dispensed to adults between January 2014 and February 2020. Of these prescriptions, 1 924 812 (2.2%) were excluded from this study owing to missing covariate data or invalid or missing data on days’ supply, and 27 100 415 (31.6%) were excluded because they were not for adults in the treatment states or control states. The characteristics of the remaining 56 607 314 prescriptions are displayed in Table 3. Among the 34 364 775 unique adults with at least 1 prescription, 18 448 788 (53.7%) were female and 15 915 987 (46.3%) were male with a mean (SD) age at the earliest fill during the study period of 44.0 (17.4) years.

**Table 3.** Characteristics of Dental Opioid Prescriptions in Adult and Child Analyses

Characteristic	Patients, No. (%)	
	Adult analysis	Child analysis
No. of opioid prescriptions	56 607 314	3 720 837
Patient group		
Treatment	38 632 619 (68.2)	2 593 892 (69.7)
Control	17 974 695 (31.8)	1 126 945 (30.3)
Patient age group, y		
0-11	0	625 757 (16.8)
12-17	0	3 095 080 (83.2)
18-25	8 567 043 (15.1)	0
26-34	10 327 409 (18.2)	0
35-44	9 728 136 (17.2)	0
45-54	10 035 631 (17.7)	0
55-64	9 867 142 (17.4)	0
≥65	8 081 953 (14.3)	0
Patient sex		
Male	25 613 980 (45.2)	1 655 215 (44.5)
Female	30 993 334 (54.8)	1 999 073 (55.5)
Census region		
Northeast	8 520 520 (15.1)	538 883 (14.5)
Midwest	13 575 481 (24.0)	807 256 (21.7)
South	21 219 756 (37.8)	1 622 630 (43.6)
West	13 291 557 (23.5)	752 068 (20.2)
Method of payment		
Commercial	34 789 200 (61.5)	2 223 764 (59.8)
Medicaid	9 190 223 (16.2)	1 265 915 (34.0)
Medicare	6 720 633 (11.9)	26 983 (0.7)
Cash	5 907 258 (10.4)	204 175 (5.5)
Opioid type		
Hydrocodone	37 763 265 (66.7)	2 200 852 (59.2)
Oxycodone	6 640 386 (11.7)	467 916 (12.6)
Codeine	9 753 053 (17.3)	886 911 (23.8)
Tramadol	2 252 644 (4.0)	66 824 (1.8)
Other^a^	197 966 (0.3)	98 334 (2.6)
Prescriber specialty		
General dentist or dental subspecialist	43 074 808 (76.1)	1 654 663 (44.5)
Oral and maxillofacial surgeon	13 535 206 (23.9)	2 066 174 (55.5)
Opioid dispensing in past 90 d		
Yes	17 229 359 (30.4)	376 086 (10.1)
No	39 377 955 (69.6)	3 344 751 (89.9)

^a^ See eAppendix 1 in Supplement 1 for a list of other opioids.

Table 1 describes the features of limits in the 22 treatment states. Among these states, 1 restricted opioid prescriptions to a 10-day supply, 14 to a 7-day supply, 3 to a 5-day supply, 1 to a 4-day supply, and 2 to a 3-day supply. The remaining state (Rhode Island) restricted opioid prescriptions to 20 doses, which corresponded to a 3- to 4-day supply depending on the frequency of opioid dosing. Limits varied by the type of prescription (eg, initial prescriptions [as defined in Table 1] vs those for acute pain) and whether the prescription was for all opioids vs opioids in specific DEA Schedules. Overall, 11 treatment states allowed dentists to exceed the duration limit based on professional judgment.

eAppendix 3 in Supplement 1 displays event study plots with and without a 6-month anticipation period. In the plot with no anticipation period, the magnitudes of preintervention coefficients began to decline approximately 5 months prior to limit enactment. When assuming a 6-month anticipation period, this decline was attenuated. Preintervention coefficients were not different from zero, suggesting that the parallel trends assumption was satisfied.

Among all prescriptions in the sample, the mean (SD) duration was 3.4 (2.2) days and median (25th-75th percentile) duration was 3.0 (2-5) days. During the study period, monthly mean days’ supply decreased from 3.4 to 3.0 days in the treatment states compared with 3.6 to 3.3 days in the control states (Figure, A). Limits were not associated with changes in the duration of dental opioid prescriptions for adults (differential change in mean days’ supply: −0.06 days; 95% CI, −0.11 to <0.001 days). The association between limit implementation and the duration of these prescriptions did not vary by limit restrictiveness or by dentist specialty (Table 4).

**Figure.**
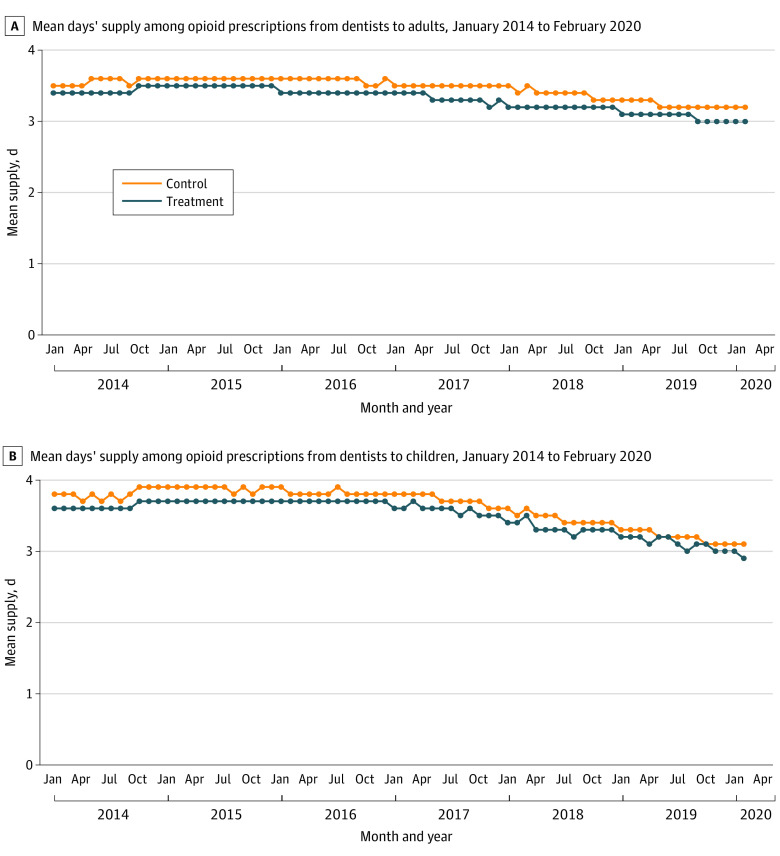
Monthly Mean Days’ Supply Among Opioid Prescriptions From Dentists in Treatment and Control States

**Table 4.** Association Between Implementation of State Opioid Prescribing Limits and Duration of Dental Opioid Prescriptions

Analysis	Differential change in mean days’ supply (95% CI)^a^	
	Adult analysis	Child analysis
Main analysis	−0.06 (−0.11 to <0.001)	−0.07 (−0.15 to 0.02)
Differential association among states restricting opioid prescriptions to ≤5-d supply vs >5-d supply	−0.06 (−0.16 to 0.05)	−0.06 (−0.14 to 0.03)
Differential association among states restricting opioid prescriptions to 3-d supply vs >3-d supply	−0.04 (−0.11 to 0.02)	−0.04 (−0.21 to 0.14)
Differential association among opioid prescriptions written by oral and maxillofacial surgeons vs other dentists	−0.11 (−0.33 to 0.11)	−0.15 (−0.31 to 0.01)
Sensitivity analysis		
No anticipation	−0.05 (−0.12 to 0.02)	−0.07 (−0.17 to 0.03)
3-mo Anticipation	−0.06 (−0.12 to <0.01)	−0.07 (0.17 to −0.02)
Doubly robust difference-in-differences estimation	−0.06 (−0.22 to 0.09)	−0.07 (−0.18 to 0.03)

^a^ Values were derived from an adjusted 2-way fixed-effects difference-in-differences model.

### Child Analysis

The IQVIA database included 5 475 704 opioid prescriptions from dentists dispensed to US children from January 2014 through February 2020. Of these prescriptions, 147 128 (2.7%) were excluded due to missing covariate data or invalid or missing data on days’ supply, and 1 607 739 (29.3%) were excluded because they were not for children in the treatment states or control states. The characteristics of the remaining 3 720 837 prescriptions are displayed in Table 3. Among the 3 165 880 unique children with at least 1 prescription, 1 740 449 (55.0%) were female and 1 425 431 (45.0%) were male, with a mean (SD) age at the earliest fill during the study period of 14.4 (3.5) years.

Table 2 describes the features of limits in the 23 treatment states in the child analysis. Among these states, 1 restricted opioid prescriptions to a 10-day supply, 14 to a 7-day supply, 4 to a 5-day supply, 1 to a 4-day supply, and 3 to a 3-day supply. Compared with the adult analysis, a greater proportion of treatment states in the child analysis restricted opioid prescriptions to a 5-day supply or less. Overall, 13 treatment states allowed dentists to exceed the duration limit based on professional judgment.

eAppendix 4 in Supplement 1 displays event study plots with and without a 6-month anticipation period. In the plot with no anticipation period, the magnitudes of preintervention coefficients began to decline approximately 6 months prior to limit enactment. When assuming a 6-month anticipation period, this decline was attenuated. Moreover, all but 1 preintervention coefficient was different from zero, suggesting that the parallel trends assumption was mostly satisfied.

Among all prescriptions in the sample, the mean (SD) duration was 3.6 (2.2) days and median (25th-75th percentile) duration was 3.0 (2-5) days. During the study period, monthly mean days’ supply decreased from 3.6 to 2.9 days in the treatment states compared with 3.9 to 3.2 days in the control states (Figure, B). As in the adult analysis, limits were not associated with changes in the duration of opioid prescriptions for children (differential change in mean days’ supply: −0.07 days; 95% CI, −0.15 to 0.02 days). Moreover, the association between limit implementation and the duration of these prescriptions did not vary by limit restrictiveness or dentist specialty (Table 4).

### Sensitivity Analysis 

For both the adult and child analyses, conclusions were unchanged when assuming a 3-month anticipation period, when assuming no anticipation period, or when using doubly robust difference-in-differences estimation. Limit implementation was associated with a decrease in the size of opioid prescriptions of −3.7 (95% CI, −5.8 to −1.7) MMEs for adults and −3.7 (95% CI, −6.9 to −0.5) MMEs for children, corresponding to a decrease of less than 1 pill containing 5 mg hydrocodone. However, event study plots suggested that the parallel trends assumption was not met, suggesting that the findings should be interpreted with caution (eAppendix 5 in Supplement 1). Finally, limit implementation was not associated with changes in the proportion of opioid prescriptions exceeding a 5-day or 7-day supply in either the adult or child analysis (eAppendix 6 in Supplement 1).

## Discussion

In this analysis, the median duration of dental opioid prescriptions dispensed to adults and children from January 2014 through February 2020 was 3.0 days. Implementation of state opioid prescribing limits, most of which allowed up to a 7-day supply of opioids, was not associated with changes in the duration of these prescriptions. These findings are consistent with those of a growing body of studies suggesting that state limits have done little to curb excessive opioid prescribing for acute pain.^8,10,20,21^

There may be at least 2 reasons for the lack of association between implementation of limits and the duration of dental opioid prescriptions. First, approximately half of the treatment states in the adult and child analyses allowed dentists to exceed the limit based on their professional judgment. Second, the duration of dental opioid prescriptions was typically below the level allowed by most limits prior to enactment of the PDMP use mandate, meaning that limits had little ability to reduce this duration in the first place. The duration of dental opioid prescriptions did not change even among states that restricted these prescriptions to a 3-day supply. Thus, even more restrictive limits, such as those allowing only a 2-day supply of opioids, may be needed to decrease the duration of dental opioid prescriptions.

While policy makers could pursue these more restrictive limits for dentists, it could be argued that the duration of dental opioid prescriptions is not the most important target for intervention. The majority of dental opioid prescriptions are written for procedures for which opioids are not more effective than nonopioids.^22,23^ One such procedure, tooth extraction, generated two-thirds of the dental opioid prescriptions in a previous study.^24^ Consequently, policy makers might prioritize interventions with a greater potential to reduce the probability of opioid prescribing by dentists in the first place. For example, PDMP use mandates, at least to the degree that dentists comply with them,^25^ may decrease this probability by increasing the hassle costs associated with writing an opioid prescription.

### Strengths and Limitations

This study has several strengths. First, we used a national all-payer prescription database (IQVIA) and a policy database created by epidemiologists (PDAPS).^14^ Second, we used a difference-in-differences design and carefully selected the treatment and control states. Finally, we assessed for heterogeneity in associations by limit restrictiveness and prescriber specialty, providing important context for the results.

The study also has several limitations. First, the study did not include states that enacted limits in 2019 and thereafter. However, each of the 4 states that implemented limits in 2019 restricted opioid prescriptions to a 7-day supply or greater,^9^ suggesting that their implementation likely was not associated with changes in the duration of dental opioid prescriptions.^9^ Second, the IQVIA database does not capture dispensing from pharmacies in facilities affiliated with the US Department of Veterans Affairs or from health system–specific pharmacies, such as those affiliated with Kaiser Permanente. Finally, because the IQVIA database lacked claims data for dental procedures, we could not assess changes in the probability of opioid prescription dispensing after such procedures. However, there is little reason to suspect these changes would occur, as opioid prescribing limits mostly target the duration of opioid prescriptions and not the decision to prescribe opioids.

## Conclusions

In this difference-in-differences cross-sectional study of national pharmacy dispensing data, state opioid prescribing limits were not associated with changes in the duration of dental opioid prescriptions from dentists for either adults or children. Future research is needed to investigate whether other interventions play a role in reducing dental opioid prescribing.

## Notes

Supplement 1. **eAppendix 1.** Opioid Analgesics Included in Analyses **eAppendix 2.** Enactment Dates of Prescription Drug Monitoring Program Use Mandates for Dentists **eAppendix 3.** Event Study Plots for the Adult Analysis **eAppendix 4.** Event Study Plots for the Child Analysis **eAppendix 5.** Event Study Plots for Sensitivity Analysis Assessing Changes in Total Morphine Milligram Equivalents **eAppendix 6.** Association Between Opioid Prescribing Limits and the Proportion of Opioid Prescriptions From Dentists That Exceeded a 5-Day and 7-Day Supply

Supplement 2.
Data Sharing Statement
