# Supplementary material for: Association Between State Opioid Prescribing Limits and Duration of Opioid Prescriptions From Dentists
Source: JAMA Netw Open. 2023 Jan 11;6(1):e2250409. doi: 10.1001/jamanetworkopen.2022.50409 (PMC9857382; doi:10.1001/jamanetworkopen.2022.50409)
Supplement: Supplement 1. — eAppendix 1. Opioid Analgesics Included in Analyses eAppendix 2. Enactment Dates of Prescription Drug Monitoring Program Use Mandates for Dentists eAppendix 3. Event Study Plots for the Adult Analysis eAppendix 4. Event Study Plots for the Child Analysis eAppendix 5. Event Study Plots for Sensitivity Analysis Assessing Changes in Total Morphine Milligram Equivalents eAppendix 6. Association Between Opioid Prescribing Limits and the Proportion of Opioid Prescriptions From Dentists That Exceeded a 5-Day and 7-Day Supply [file jamanetwopen-e2250409-s001.pdf]

Supplemental Online Content

Chua K-P, Nguyen TD, Waljee JF, Nalliah RP, Brummett CM. Association between state opioid prescribing limits and duration of opioid prescriptions from dentists. *JAMA Netw Open*. 2023;6(1):e2250409. doi:10.1001/jamanetworkopen.2022.50409

eAppendix 1. Opioid Analgesics Included in Analyses

eAppendix 2. Enactment Dates of Prescription Drug Monitoring Program Use Mandates for Dentists

eAppendix 3. Event Study Plots for the Adult Analysis

eAppendix 4. Event Study Plots for the Child Analysis

eAppendix 5. Event Study Plots for Sensitivity Analysis Assessing Changes in Total Morphine Milligram Equivalents

eAppendix 6. Association Between Opioid Prescribing Limits and the Proportion of Opioid Prescriptions From Dentists That Exceeded a 5-Day and 7-Day Supply

This supplemental material has been provided by the authors to give readers additional information about their work.

© 2023 Chua K-P et al. *JAMA Network Open.*

eAppendix 1. Opioid analgesics included in analyses

Opioid analgesics included benzhydrocodone, buprenorphine, butorphanol, codeine, dihydrocodeine, fentanyl, hydrocodone, hydromorphone, levorphanol, meperidine, methadone, morphine, nalbuphine, opium, oxycodone, oxymorphone, pentazocine, propoxyphene, sufentail, tapentadol, and tramadol. We limited to opioids with the following routes of administration: oral (including liquids), transdermal (e.g., patches), sublingual, nasal, rectal, and buccal. We did not include buprenorphine formulations indicated for opioid use disorder or opioid cough-and-cold medications.

© 2023 Chua K-P et al. *JAMA Network Open.*

eAppendix 2. Enactment dates of prescription drug monitoring program use mandates for dentists

We obtained information on PDMP use mandates through a database created for our team by the Prescription Drug Abuse Policy System. We recorded whether a state implemented a PDMP use mandate that included dentists for the first time or whether it amended an existing PDMP use mandate so that it newly included dentists.

**State**	**Effective date of PDMP use mandate including dentists**
**TREATMENT STATES**	
Arizona	10/1/2017
Arkansas	8/1/2017
Colorado	5/21/2018[a]
Connecticut	10/1/2015
Indiana	11/1/2014
Kentucky	7/20/2012
Louisiana	1/20/2018
Massachusetts	12/5/2014
Michigan	6/1/2018
Minnesota	No mandate
Mississippi	No mandate
Missouri	No mandate
New Jersey	11/1/2015
Nebraska*	No mandate
New York	8/27/2013
North Carolina	6/25/2018
Ohio	4/1/2015
Oklahoma	11/1/2015
Pennsylvania*	6/30/2015
Rhode Island*	6/28/2016
South Carolina	5/29/2017
Virginia	7/1/2015
Vermont	No mandate
West Virginia	6/10/2016[b]
**CONTROL STATES**	
Alabama	9/9/2018
California	1/1/2017
DC	No mandate
Georgia	7/1/2018
Idaho	No mandate
Iowa	7/1/2018
Kansas	No mandate
New Mexico	9/28/2012
North Dakota	1/1/2018
Oregon	No mandate
South Dakota	No mandate
Wisconsin	4/1/2017

* - NE and PA were only treatment states in the child analysis; RI was only a treatment state in the adult analysis

[a]Colorado's PDMP use mandate was enacted the same date as the opioid prescribing limit, but it only applied to second opioid prescriptions. Given this limited scope, we opted to retain Colorado in both analyses.

[b]On June 7, 2018, the same that an opioid prescribing limit was implemented, West Virginia amended its PDMP use mandate to change the type of prescriptions affected. However, the mandate applied to all opioids both before and after this amendment. Consequently, we opted to retain West Virginia in both analyses. We considered the effective date of the PDMP use mandate for dentists to be 6/10/2016, not 6/7/2018.

© 2023 Chua K-P et al. *JAMA Network Open.*

eAppendix 3. Event study plots for the adult analysis

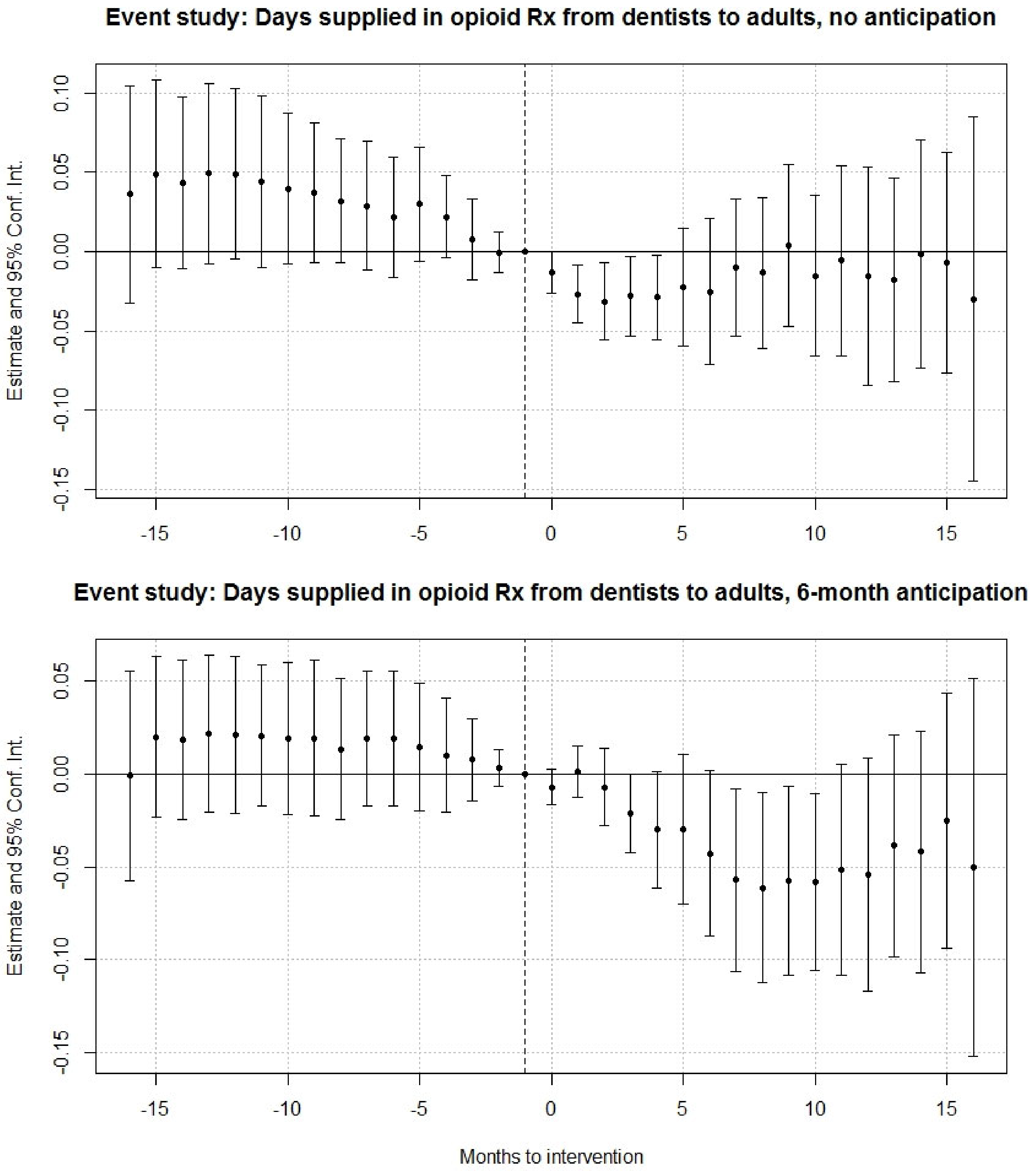

© 2023 Chua K-P et al. *JAMA Network Open.*

eAppendix 4. Event study plots for the child analysis

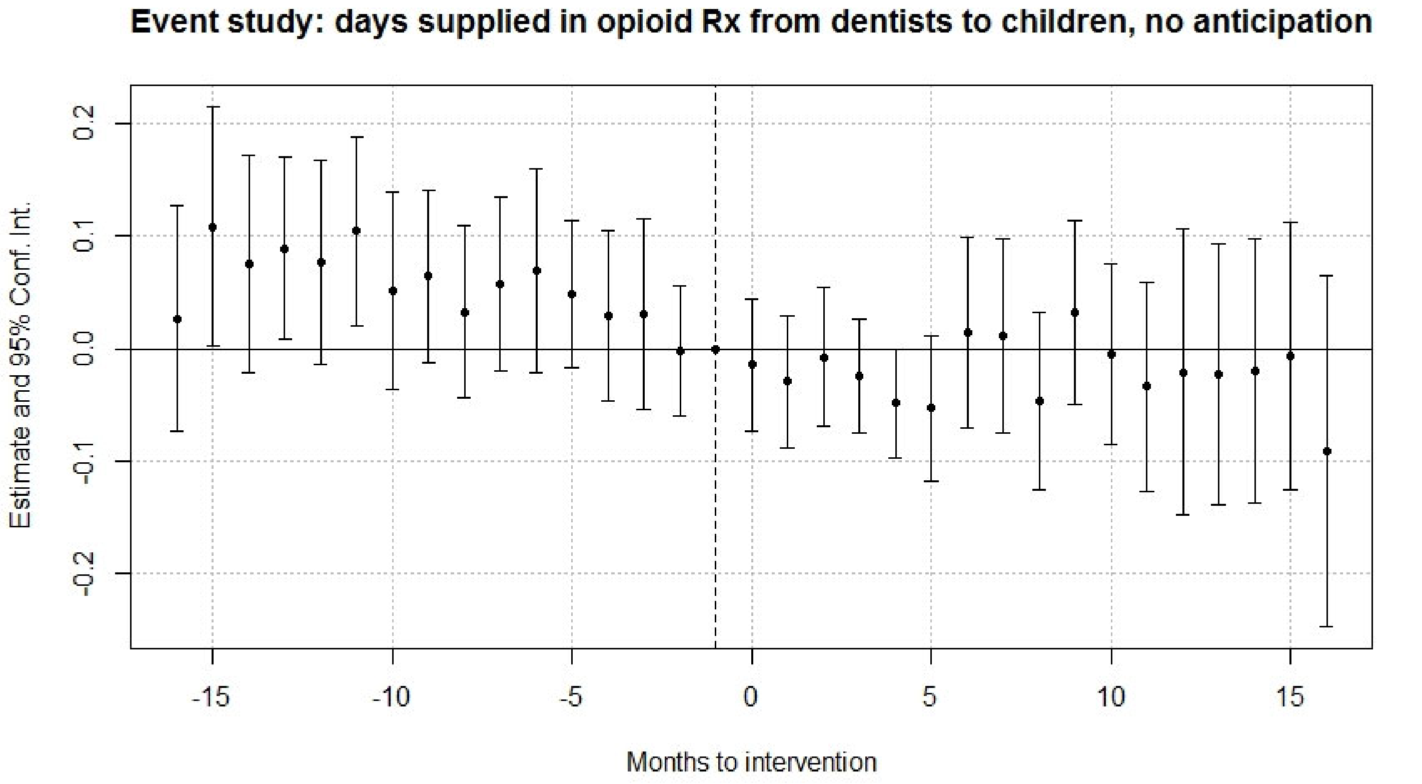

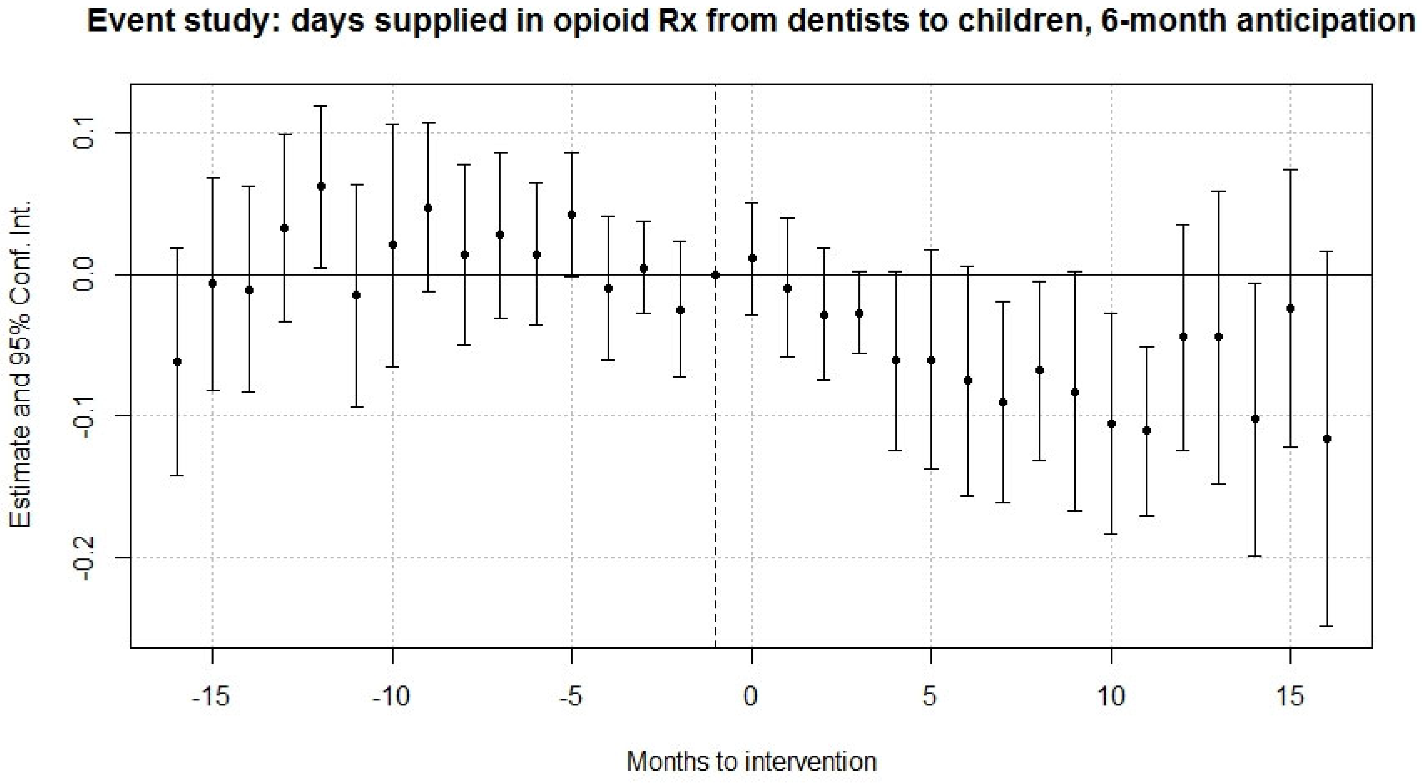

© 2023 Chua K-P et al. *JAMA Network Open.*

eAppendix 5. Event study plots for sensitivity analysis assessing changes in total morphine milligram equivalents

When modeling total MME, there was still some evidence of anticipation in the adult analysis even when including a 6-month anticipation period. Moreover, some pre-intervention coefficients were different from zero in the child analysis. These findings suggest that the parallel trends assumption was violated to a greater degree compared with our main analysis.

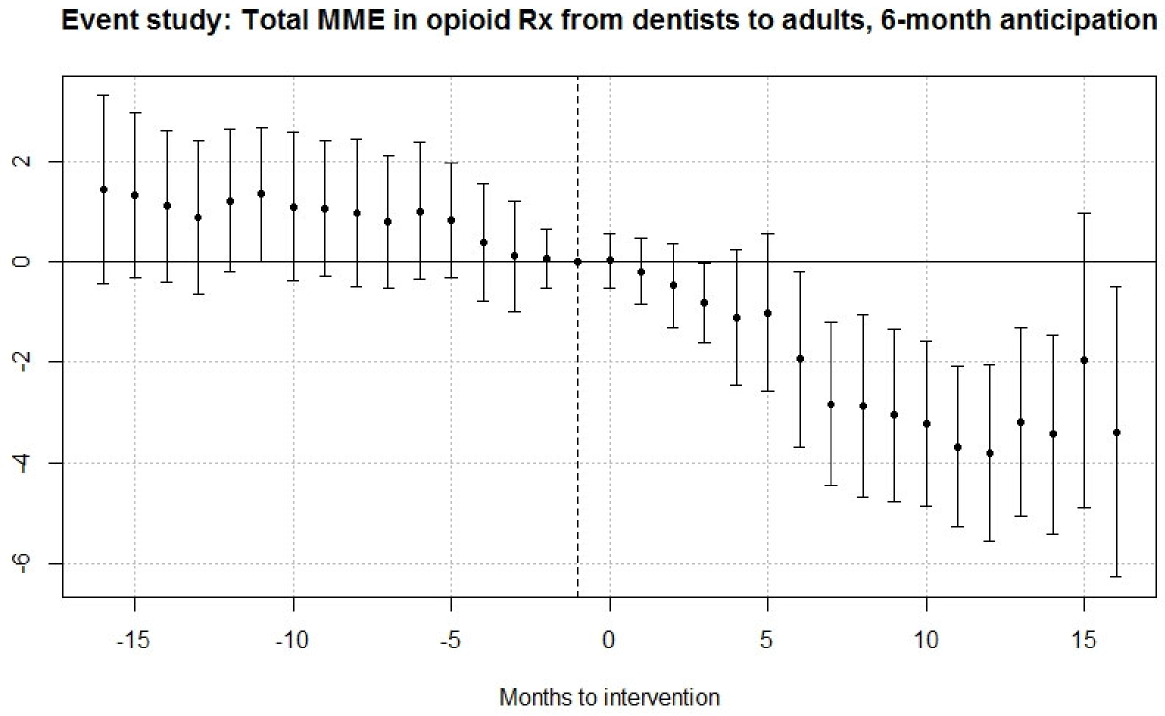

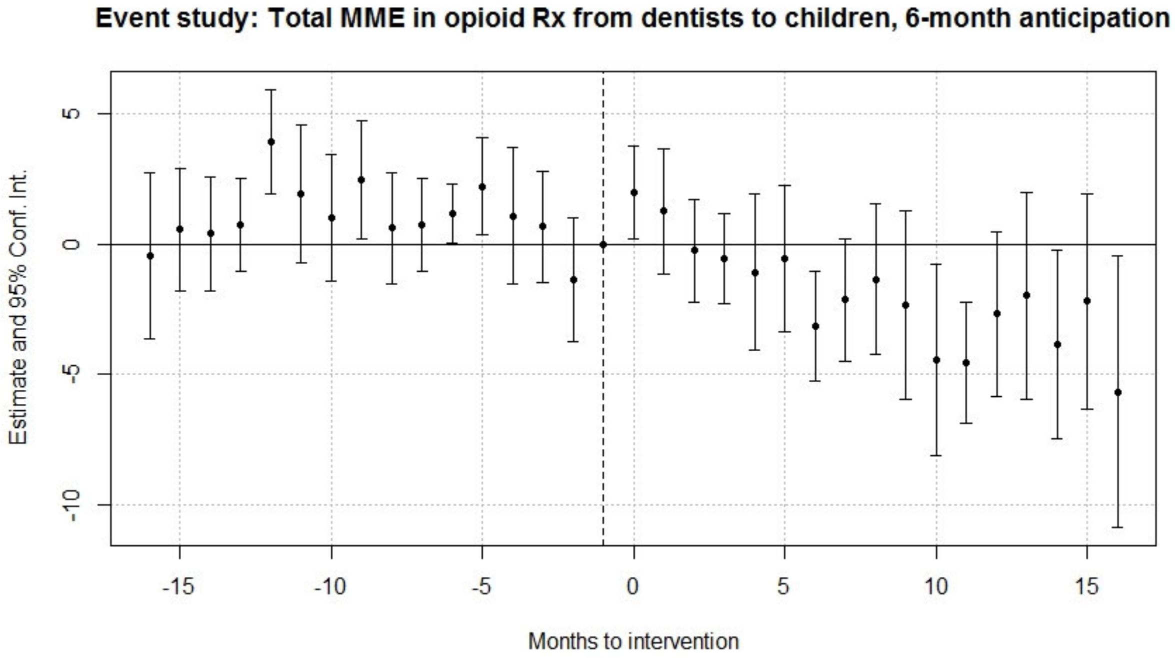

© 2023 Chua K-P et al. *JAMA Network Open.*

eAppendix 6. Association between opioid prescribing limits and the proportion of opioid prescriptions from dentists that exceeded 5- and 7-day supply.

Outcome	Adult analysis	Child analysis
% Rx > 5-day supply	-0.3 (-0.7, 0.12)	-0.3 (-1.2, 0.5)
% Rx > 7-day supply	-0.07 (-0.4, 0.2)	-0.2 (-0.6, 0.2)

© 2023 Chua K-P et al. *JAMA Network Open.*
