# Supplementary material for: Association Between State Opioid Prescribing Limits and Duration of Opioid Prescriptions From Dentists
Source: JAMA Netw Open. 2023 Jan 11;6(1):e2250409. doi: 10.1001/jamanetworkopen.2022.50409 (PMC9857382; doi:10.1001/jamanetworkopen.2022.50409)
Supplement: Supplement 2. — Data Sharing Statement [file jamanetwopen-e2250409-s002.pdf]

Data Sharing Statement

Chua. Association Between State Opioid Prescribing Limits and Duration of Opioid Prescriptions From Dentists. *JAMA Netw Open*. Published January 11, 2023. doi:10.1001/jamanetworkopen.2022.50409

Data
Data available: No

Additional Information
Explanation for why data not available: Data are proprietary
